# Developmental Foundations of Psychosocial Interventions in Pediatric Oncology: A Lifespan Framework for Resilience

**DOI:** 10.3390/children13020198

**Published:** 2026-01-30

**Authors:** Antonios I. Christou, Georgia Kalfadeli, Flora Bacopoulou

**Affiliations:** 1 Department of Special Education, University of Thessaly, 38221 Volos, Greece; gkalfadeli@uth.gr; 2 Clinic for Assessment of Adolescent Learning Difficulties, Center for Adolescent Medicine and UNESCO Chair in Adolescent Health Care, First Department of Pediatrics, Medical School, National and Kapodistrian University of Athens, 11527 Athens, Greece; fbacopoulou@med.uoa.gr

**Keywords:** adolescence, cancer survivorship, child development, cognitive–behavioral therapy, emerging adulthood, pediatric oncology, psychosocial interventions, resilience, school re-entry, developmental stages

## Abstract

**Highlights:**

**What are the main findings?**

- Targeted psychosocial interventions that take into account the developmental stage (such as therapeutic play, cognitive–behavioral techniques, and school reintegration programs) significantly improve the emotional regulation and school adjustment of children with cancer.
- The effectiveness of interventions varies by age and type of cancer, with interventions that involve family and peers having the most consistent positive outcomes.

**What are the implications of the main findings?**

- Developing interventions based on developmental models can enhance the long-term mental resilience and academic success of child survivors.
- More cross-cultural and long-term research is needed to determine intervention models that can be widely applied in pediatric oncology programs.

**Abstract:**

Background/Objectives: In recent years, improvements and innovative treatments in pediatric cancer have significantly increased survival rates, but challenges in both cognitive and psychosocial development in children remain significant. This review applies a comprehensive framework to evaluate psychosocial interventions in pediatric populations, offering novel insights into intervention strategies and their effectiveness across diverse contexts. Methods: A systematic search was conducted in the PubMed, Scopus, PsycINFO, and Web of Science databases for the period 2000–2024. Controlled studies, systematic reviews, and qualitative studies examining psychosocial interventions for children and adolescents with cancer or survivors were included. Quality assessment was performed using the RoB2 tool, and data were analyzed using narrative synthesis by age group and type of intervention. Results: Studies have shown that developmentally targeted interventions, such as therapeutic play, cognitive–behavioral therapy, and school reintegration programs, improve emotional regulation, cognitive functioning, and social adjustment in children with cancer. However, the heterogeneity of the samples and the variety of measurements limit the generalizability of the results. Conclusions: Integrating a developmental perspective into the design of psychosocial interventions can enhance their effectiveness and sustainability in pediatric oncology. Future research should focus on long-term, culturally sensitive programs and their implementation in clinical practice.

## 1. Introduction

Advances in pediatric cancer treatment over the past decades have significantly increased survival rates, transforming childhood cancer into a chronic condition with long-term developmental implications [1]. However, survival does not always equate to full recovery. Many children and adolescents continue to experience difficulties in cognitive functioning, emotional regulation, and social adjustment, which may disrupt school performance, daily functioning, and overall quality of life [2]. These challenges often persist well beyond the completion of treatment, necessitating systematic monitoring and ongoing psychosocial support [3].

Cognitive difficulties are among the most frequently reported long-term effects, particularly in children exposed to neurotoxic treatments such as cranial irradiation or high-dose methotrexate. These may include deficits in attention, working memory, processing speed, and executive functions, which can interfere with academic achievement and learning acquisition [4,5]. Emotional difficulties also commonly arise, as children must cope with fear, uncertainty, and distress during diagnosis and treatment [6]. Symptoms of anxiety, depression, and heightened stress responses may persist into survivorship, often accompanied by worries about relapse or challenges in regaining a sense of normalcy [7,8]. Social reintegration can be equally demanding, with many children facing isolation, reduced peer participation, altered self-image, and difficulties re-establishing friendships following long periods of hospitalization or treatment-related fatigue [9].

These cognitive, emotional, and social disruptions intersect to influence the child’s developmental trajectory. Importantly, the type and intensity of difficulties vary across developmental stages [10,11]. Preschool-aged children often rely on play and caregiver co-regulation to process medical experiences, whereas school-aged children face challenges related to academic reintegration and growing cognitive demands. Adolescents, in turn, must navigate issues of identity formation, autonomy, social belonging, and future planning while simultaneously coping with the impacts of cancer [12,13,14]. Tailoring psychosocial support to the developmental needs of each age group is therefore essential. In addition, family cohesion, parental mental health, and the school environment play a critical role in shaping resilience, highlighting the need for interventions that extend beyond the individual child [15,16].

Despite the substantial literature on the late effects of pediatric cancer, current knowledge remains fragmented. Most existing studies examine cognitive, emotional, or social outcomes separately, and few reviews integrate these domains within a developmental framework [17]. Moreover, there is limited synthesis of which psychosocial interventions are most effective at different developmental stages, and the mechanisms linking neurocognitive vulnerabilities with psychosocial resilience remain insufficiently explored [18,19]. This fragmentation makes it challenging for clinicians to identify age-appropriate, evidence-based strategies for supporting children and adolescents across the cancer trajectory. Rather than organizing the evidence according to cancer type or treatment modality, this review adopts a developmental, age-based framework. This approach allows for the examination of psychosocial interventions in relation to children’s evolving cognitive and emotional capacities, highlighting mechanisms of resilience across developmental stages. Despite the growing body of literature on psychosocial outcomes and interventions in pediatric oncology, existing reviews tend to focus either on isolated cognitive or emotional outcomes, specific diagnostic groups, or single types of interventions. Moreover, few reviews adopt a developmental perspective that systematically examines how intervention needs and effectiveness vary across childhood and adolescence. As a result, there remains a limited synthesis of how age-sensitive psychosocial interventions jointly support cognitive, emotional, and social adaptation over time. The present review addresses these gaps by applying a developmental, lifespan-oriented framework to evaluate psychosocial interventions across distinct age groups in pediatric oncology.

Therefore, the present review has the following aims:

(1) Synthesize evidence on cognitive, emotional, and social outcomes as examined in intervention-based studies targeting children and adolescents with cancer;
(2) Examine psychosocial interventions through a developmental lens;
(3) Compare intervention effectiveness across developmental stages;
(4) Identify key risk and resilience factors across childhood and adolescence;
(5) Propose a lifespan developmental framework to guide clinical practice in pediatric oncology.

## 2. Materials and Methods

### 2.1. Study Design

This study follows the narrative review methodology and is combined with a conceptual framework synthesis. The selected time frame (2000–2024) reflects the period during which structured psychosocial and neurocognitive interventions in pediatric oncology became more systematically developed and empirically evaluated. This methodology was chosen because it allows for a broad, descriptive, and combinatorial representation of the existing literature while also allowing for the synthesis of theoretical and empirical findings. The overall objective of choosing this methodology is to develop a developmental theoretical framework for psychosocial interventions in pediatric oncology.

### 2.2. Literature Search Strategy

Searches were performed in PubMed, Scopus, PsycINFO, and Web of Science from January 2000 to December 2024. The search strategy combined free-text keywords and Medical Subject Headings (MeSH) terms focusing on pediatric cancer, child and adolescent development, psychosocial care, resilience, executive functions, emotional regulation, and family or school-based support systems. To ensure transparency and reproducibility, the complete search strings adapted to the indexing conventions and Boolean operators of each database are presented below.

PubMed:

("pediatric cancer"[Title/Abstract] OR "childhood cancer"[Title/Abstract] OR "pediatric oncology"[Title/Abstract])

AND ("development"[Title/Abstract] OR "child development"[Title/Abstract] OR "adolescent development"[Title/Abstract] OR "developmental outcomes"[Title/Abstract])

AND ("psychosocial interventions"[Title/Abstract] OR "psychosocial support"[Title/Abstract] OR "psychological care"[Title/Abstract] OR "behavioral interventions"[Title/Abstract] OR "resilience"[Title/Abstract] OR "protective factors"[Title/Abstract])

AND ("2000/01/01"[Date—Publication] : "2024/12/31"[Date—Publication])

Scopus:

TITLE-ABS-KEY("pediatric cancer" OR "childhood cancer" OR "pediatric oncology") AND TITLE-ABS-KEY("development" OR "child development" OR "adolescent development" OR "developmental outcomes")

AND TITLE-ABS-KEY("psychosocial intervention" OR "psychosocial support" OR "psychological care" OR "behavioral intervention*" OR "resilience")

AND (PUBYEAR > 1999 AND PUBYEAR ≤ 2024)

PsycINFO (via APA PsycNet):

DE “Childhood Cancer” OR DE “Pediatric Oncology”

AND (DE "Child Development" OR DE "Adolescent Development" OR DE "Developmental Outcomes")

AND (DE "Psychosocial Intervention" OR DE "Psychosocial Support" OR DE "Psychological Services" OR DE "Behavioral Interventions" OR DE "Resilience" OR DE "Protective Factors")

AND PEER(yes) AND Year ≥ 2000 AND Year ≤ 2024

Web of Science (Core Collection):

TS = ("development" OR "child development" OR "adolescent development" OR

"developmental outcomes")

AND TS = ("psychosocial intervention" OR "psychosocial support" OR

"psychological care" OR "behavioral intervention" OR

"resilience") Refined by: DOCUMENT TYPES (Article OR Review) AND LANGUAGES (English) Timespan: 2000–2024

### 2.3. Inclusion/Exclusion Criteria

The inclusion criteria were established to ensure that the review focused on high-quality empirical evidence relevant to the cognitive, emotional, and social functioning of children and adolescents with cancer. Eligible studies were those published between January 2000 and December 2024 and conducted with participants aged 4 to 18 years, diagnosed with any form of pediatric cancer, and either undergoing treatment or in survivorship. Only peer-reviewed empirical research articles were considered, including randomized controlled trials, longitudinal studies, cross-sectional designs, mixed-methods investigations, and intervention-based research. Studies were required to examine at least one of the following outcome domains: cognitive functioning (attention, working memory, processing speed, executive functions), emotional or psychological well-being (anxiety, depression, stress, emotional regulation), or social functioning (peer relationships, school reintegration, social participation). Although studies were eligible if they reported outcomes in at least one domain (cognitive, emotional, or social), studies focusing exclusively on cognitive or academic outcomes without psychosocial relevance were excluded. To be eligible, studies also needed to evaluate psychosocial, cognitive–behavioral, educational, or multidisciplinary interventions targeting children or adolescents with cancer. Records were automatically excluded if they were non-empirical publications (e.g., reviews, editorials), included adult-only samples, were not intervention-based, or were not published in English. Additional exclusions at the full-text screening stage were based on the lack of relevant cognitive, emotional, or psychosocial outcomes. Only full-text articles available in English were included.

Exclusion criteria were applied to maintain conceptual and methodological clarity. Studies focusing on adults with cancer, or mixed samples in which outcomes specific to children could not be isolated, were excluded. Review papers, systematic reviews, meta-analyses, theoretical articles, commentaries, editorials, conference abstracts, and dissertations were not included, as the aim of this review was to synthesize primary empirical evidence. Studies that focused exclusively on medical or biological outcomes without assessing cognitive, emotional, or social variables were also excluded. Single-case studies, reports with insufficient methodological detail, and articles not available in full text or published in languages other than English were omitted.

Title/abstract screening and full-text review were conducted independently by two reviewers. Disagreements were resolved through discussion until a consensus was reached. Risk of bias was assessed using study-design-appropriate tools (RoB 2 for randomized trials and relevant quality appraisal tools for observational studies).

The process of identifying and selecting studies followed the PRISMA standard. A total of 836 records were retrieved from databases (*n* = 4) and registries (*n* = 832), including 278 from PubMed, 227 from Scopus, 192 from PsycINFO, and 135 from Web of Science, of which 94 duplicates, 12 records that were automatically rejected, and 27 that were removed for other reasons were excluded before the initial screening. Automatic exclusions included non-empirical publications (e.g., editorials, protocols, case reports), studies not involving pediatric cancer populations, and records without sufficient methodological information. Records removed for other reasons included articles with inaccessible full texts or outcomes unrelated to intervention effects. Thus, 699 records proceeded to the screening phase, where 496 were excluded based on title and abstract. Of the 203 records requested for retrieval, 16 were unavailable, resulting in 187 studies being assessed for eligibility. At this stage, 61 studies focusing exclusively on adults, 36 that did not include emotion/sensation-related outcomes, and 18 that were non-experimental in nature were excluded. Finally, 72 new studies met the criteria and were included in the review.

### 2.4. Data Extraction and Synthesis

**Figure 1:**
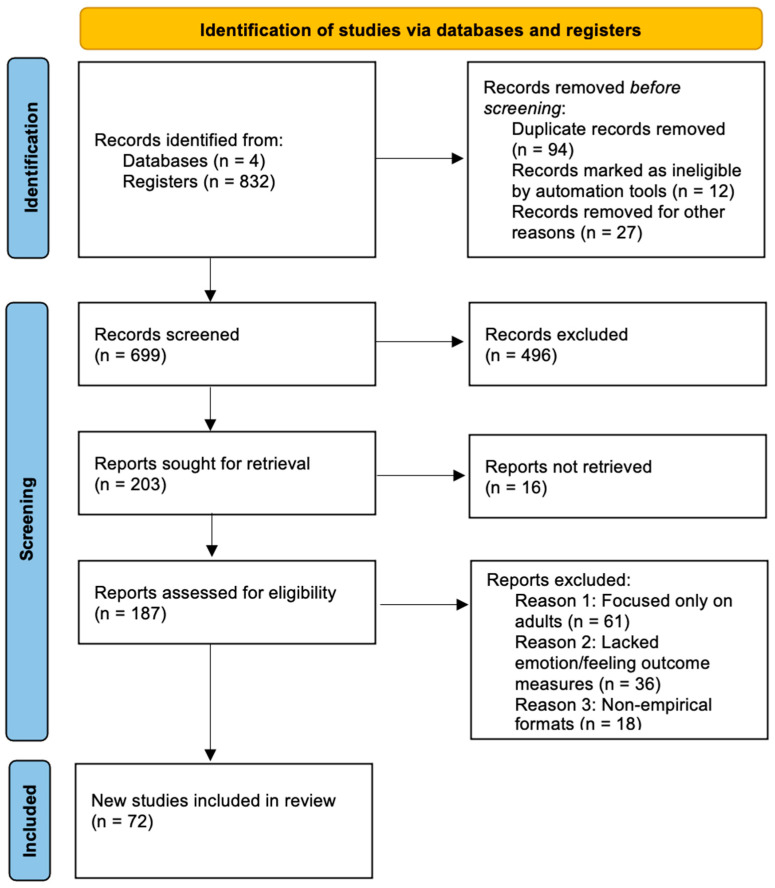
PRISMA-style flow diagram of study selection.

Data extraction was performed using a standardized recording form developed for the purposes of this review. For each study, information was collected on the study design, sample characteristics (age, stage of treatment or survival, type of cancer), a description of the psychosocial intervention or protective factors and developmental framework, assessment tools, and main findings. The extraction process was performed by two independent evaluators, with differences resolved through discussion or a third reviewer (see Figure 1).

Data synthesis was performed using a narrative and thematic approach, taking into account the heterogeneity of the studies in terms of samples, interventions, and outcomes. Findings were grouped by developmental period, type of psychosocial intervention, and outcome category, with a particular focus on resilience mechanisms and factors influencing the implementation and effectiveness of interventions. The quality of the studies was taken into account when interpreting the results, and potential sources of bias were noted. The synthesized findings informed the subsequent development of a conceptual framework integrating developmental theory with empirical evidence. Age group was prioritized over pathology as the primary organizing principle in order to reduce clinical heterogeneity and better capture developmentally specific intervention effects across cognitive and emotional domains.

### 2.5. Conceptual Framework Development

The conceptual framework was developed based on a combined analysis of theoretical models of development (neurodevelopmental processes and socio-emotional maturation) and empirical findings from the studies included in the review. Initially, the data were organized by developmental period (early childhood, middle childhood, adolescence) and by type of outcome (developmental skills, psychosocial adjustment). At the same time, the interventions were classified according to their basic characteristics and possible mechanisms of action (emotion regulation, family support, and resilience programs).

Next, common patterns were identified between developmental needs and elements of psychosocial interventions in order to highlight the protective factors and resilience mechanisms that operate differently at each age stage. The final conceptual framework illustrates the dynamic relationship between developmental maturity and response to psychosocial interventions, proposing a coherent, longitudinal approach to resilience in pediatric oncology. Most studies did not compare multiple intervention types within the same developmental stage. Instead, similar intervention approaches were repeatedly applied within specific age groups, such as attachment-focused parent interventions in early childhood, allowing resilience-related processes to be inferred from recurring targets and outcomes rather than direct comparisons.

## 3. Results

The results are structured developmentally and analytically, presenting evidence on cognitive, psychosocial, and combined interventions, followed by an examination of age-specific response patterns and moderating factors that shape intervention effectiveness. Across developmental stages, most studies did not directly compare different intervention modalities within the same age group. As such, developmental patterns were derived from cross-study consistency in intervention targets and outcomes rather than head-to-head comparisons. A total of 72 studies were included in the review. Of these, 18 evaluated cognitive interventions exclusively, 32 focused on psychosocial or emotional interventions, 9 applied combined protocols, and 13 were implemented in school or community settings. The majority of studies were pilot or non-randomized interventions, while only a small percentage included follow-ups longer than 6 months. The samples ranged from small groups (*n* < 40) to medium-scale interventions (*n* = 80–120), with significant heterogeneity in terms of the age of the participants.

### 3.1. Cognitive Interventions

Studies evaluating cognitive interventions reported consistent improvement in specific dimensions of executive functions, such as working memory, cognitive control, and inhibition [20,21]. Working memory training programs (e.g., Cogmed) led to small-to-moderate increases in performance on neuropsychological tasks, as noted in more than half of the studies. In a subset of studies, gains remained stable at immediate retest but declined at 3–6 month follow-up, suggesting reduced maintenance without continued practice [22,23]. In children who had received intensive treatment regimens (e.g., brain radiation or high-dose chemotherapy), improvements were smaller, particularly on measures of processing speed.

In contrast, children who had completed their treatment at a younger age were more likely to show improvement, which the researchers themselves attributed to increased neuroplasticity [24,25]. The duration and frequency of interventions were also decisive factors: programs lasting >6 weeks and with continuous monitoring produced more sustainable improvements compared to short-term interventions [26]. In interventions that combined cognitive training with school guidance or educational support, children showed improvements in functional skills, such as performing daily school tasks, taking notes, and organizing activities. However, in programs that remained exclusively laboratory-based, the effects were limited to laboratory tests, without generalization to real-life behavior [27].

**Figure 2:**
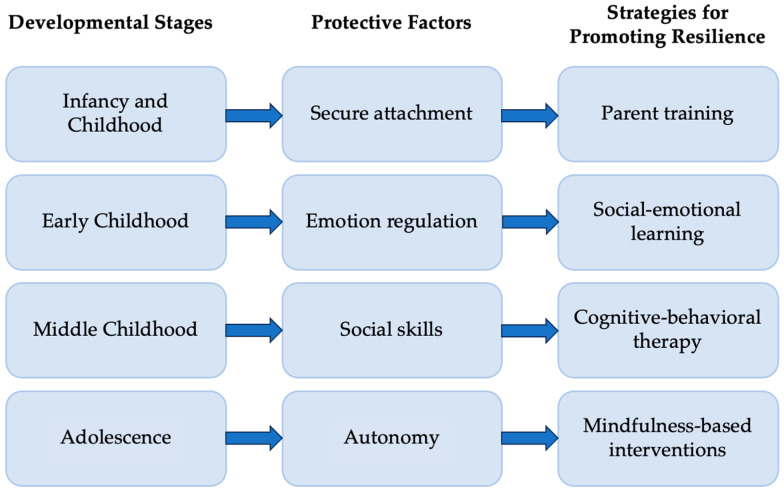
Illustration of developmental stages and strategies for strengthening resilience in children and adolescents.

In summary, cognitive interventions showed small-to-moderate improvements in measures of working memory, attention, and executive control, with the most consistent findings reported in school-aged children. The generalization of results to everyday functioning was limited, particularly in short-term interventions or those without ongoing practice. While a subset of studies employing repeated-measures designs suggested that improvements in cognitive functioning were followed by reductions in emotional distress, the evidence for a temporal or predictive relationship remains limited. Therefore, the proposed sequence whereby cognitive gains facilitate subsequent emotional adjustment should be interpreted as a developmentally informed, integrative hypothesis rather than a consistently demonstrated causal pathway (see Figure 2).

### 3.2. Psychosocial Interventions: Developmental Foundations and Resilience

In psychosocial interventions, the effect was mainly observed in indicators of emotional regulation, stress management, and social interaction [28]. Interventions that combined training in stress coping strategies with social skills enhancement showed moderate-to-high effectiveness, especially in school-aged children. Group support programs, whether in a school setting or a clinical setting, contributed to improving the sense of belonging, reducing isolation, and strengthening trust in peers [29]. For example, cognitive–behavioral interventions typically included structured sessions targeting emotional expression, coping skills, and problem-solving, while play-based interventions emphasized symbolic play, emotional labeling, and parent–child interaction. In studies evaluating play-based therapeutic interventions, children aged 3–6 years showed a significant reduction in undesirable emotional reactions, such as anxiety and anger outbursts [30]. This effect was more pronounced when parents actively participated in the intervention, which reinforced the transfer of skills to the home. Peer-support programs for adolescents showed mixed results. In groups with consistent participation and structured meetings, adolescents reported small to moderate improvements in feelings of cohesion and acceptance [31,32]. In contrast, programs with low participation or no repeated commitment did not lead to significant changes. In social–emotional interventions, the effect was stronger in preschool and school-aged children, with moderate-to-high improvement in emotional self-regulation and social skills [33]. In adolescents, individual interventions were less consistent, while group or peer-based protocols were more effective when meetings were repeated and consistent [34,35]. Across developmental stages, most psychosocial studies compared structured, developmentally targeted interventions to standard care, waitlist controls, or minimal-support conditions. Direct comparisons between different intervention types within the same age group were rare. Consequently, conclusions regarding the developmental mechanisms of resilience were drawn from cross-study consistency in targeted processes rather than head-to-head intervention comparisons.

#### 3.2.1. Infancy and Toddlerhood (0–2 Years)

In infants and toddlers, resilience is built primarily through secure relationships with parents and a stable environment. Psychosocial interventions focus on parental guidance in responding to the child’s needs, supporting early emotional regulation, and creating secure attachments. Strengthening these early bonds is a foundation for future resilience development [36].

#### 3.2.2. Early Childhood (3–5 Years)

In early childhood, resilience development is related to the child’s ability to manage their emotions and interact positively with peers. Interventions include play therapy, social skills activities, individual counseling, and active parental involvement. The integration of coping strategies and the practice of emotional self-regulation reinforce the development of protective mechanisms against stress and treatment-related challenges [37].

#### 3.2.3. Middle Childhood (6–11 Years)

In middle childhood, resilience is promoted through the development of social skills, self-regulation, and problem-solving skills. Psychosocial interventions include social skills groups, counseling, and educational activities that enhance autonomy and peer interaction. Active family involvement remains important for generalizing skills, while improving self-management and school adjustment enhances the child’s resilience to difficulties [38].

#### 3.2.4. Adolescence (12–18 Years Old)

In adolescence, the development of resilience is linked to the ability to self-regulate, socialize, and become independent. Interventions include mindfulness, individual counseling, support groups, and stress management strategies [39]. Family involvement combined with strengthening individual coping strategies allows adolescents to manage emotional difficulties, social challenges, and academic demands. Interventions that incorporate developmentally appropriate elements and protective strategies appear to significantly enhance resilience in this age group [40].

The effectiveness of psychosocial interventions for promoting resilience varies by developmental stage. In the first stage (0–2 years), the emphasis is on establishing secure relationships with parents [41]. In early childhood (3–5 years), the priority is learning social skills and emotional self-regulation [42]. In middle childhood (6–11 years), self-regulation, social participation, and problem-solving are reinforced, while in adolescence (12–18 years), attention is focused on independence, social integration, and stress management [43]. Interventions that take into account the developmental needs of each age group appear to be more effective in strengthening protective factors and promoting resilience in children and adolescents after cancer.

### 3.3. Combined Approaches to Cognitive and Psychosocial Support

Studies evaluating combined interventions consistently showed more stable and broader results than those evaluating single-dimensional interventions. Programs that incorporated executive function training (working memory, cognitive control, processing speed) with emotional regulation and social skills enhancement showed consistency in findings, both in performance tests and in functional indicators of daily behavior [44,45]. In clinical and school settings, children who participated in such programs showed increased concentration on multiple stimuli, improved ability to plan activities, and reduced difficulties in performing complex tasks [46]. In studies with follow-up periods of 3–12 months, combined interventions showed less skill loss than single-topic interventions, with higher sustainability in functional outcomes (school organization, group participation, social initiative). This stability was recorded across more than one age range.

At the same time, there was an increase in social participation, with a reduction in avoidance of group activities and improved emotion recognition, which is reflected in greater willingness to cooperate and increased emotional stability [47]. In most studies, the generalization of skills from the intervention setting to everyday life was more pronounced than in purely cognitive or exclusively psychosocial approaches. School adjustment, as rated by teachers or intervention teams, showed steady improvement, while learning difficulties and the use of academic accommodations decreased over the medium-term follow-up period. In addition, increased levels of self-confidence were observed, as reflected in self-reported questionnaires and third-party observations [48].

In some programs, the interaction between the two components of the intervention was sequential: improvement in executive functioning was associated with reduced emotional reactivity, which in turn facilitated participation in social contexts and school demands [49]. These data were recorded in repeated measurements in both laboratory tests and parent/teacher rating scales. The maintenance of results was also more stable. In studies with a follow-up of 3–12 months, children who had received combined intervention showed a smaller decline in skills compared to groups that received only cognitive or psychosocial support. This pattern was observed regardless of cancer type or treatment regimen, with greater consistency in children aged 8–12 years, where interventions were implemented in a structured setting (school or organized group) [50]. Finally, it was observed that combined interventions reduce within-group variability, i.e., the difference between children with similar medical histories. In contrast to purely cognitive programs, where the response varied greatly depending on the profile of executive functions, combined approaches had more homogeneous results, which is reflected in the stability of behavioral measurements and smoother reintegration into school and social environments [51].

### 3.4. Summary of Findings

The overall findings of the review converge on the fact that childhood cancer survivors experience both cognitive difficulties (mainly in executive functions) and psychosocial challenges (anxiety, reduced social participation) at higher levels than their peers. A comparative analysis of studies shows that children and adolescents who have survived cancer often exhibit converging patterns of difficulties in two key areas: (a) cognitive performance and (b) psychosocial functioning. In the cognitive domain, most studies highlight deficits in working memory, difficulties in inhibiting impulsive responses, and reduced processing speed, compared to their peers [52]. These findings were evident in both clinical tests and academic assessments, particularly in children who had undergone more intensive treatment protocols, while the presence of neurotoxic agents (e.g., CNS radiation) was associated with higher rates of cognitive deficits.

On the psychosocial axis, most studies reported increased levels of anxiety, emotional instability, and lower levels of social participation, especially in the first few years after completing treatment. In terms of social functioning indicators, difficulties in participating in group activities, avoidance of interaction, and increased dependence on adults or close family members were frequently observed. Repeated findings from self-report scales and third-party assessments confirmed the same pattern: reduced self-confidence, lower perception of academic ability, and feelings of social isolation, especially in adolescents [53]. Cognitive enhancement interventions linked improvement in specific executive function indicators with modest but systematic progress in daily activity performance. Several studies reported an increased ability to organize schoolwork, fewer errors in complex tasks, and better resilience to cognitive load, with greater stability in school-aged children [53,54]. At the same time, psychosocial interventions had a positive effect in areas such as empathy, emotional self-regulation, and participation in peer groups, with fewer episodes of isolation and avoidance [55].

Combined interventions were the point of maximum synergy between the two areas: children showed improvement in both cognitive indicators and behavioral/functional outcomes, which was recorded across a wider age range [56]. This improvement was not limited to laboratory measurements but was reflected in school adjustment, reactivation of social networks, and reduction in functional barriers in everyday life. Furthermore, in most long-term assessments, children who participated in combined interventions showed less skill loss compared to other forms of intervention, indicating higher sustainability of results (See Table 1).

### 3.5. Cross-Study Variability and Moderating Factors

The findings of the studies show significant variation in terms of the intensity and duration of the results. Cognitive enhancement interventions were more effective in children who were younger at the time of treatment, while the benefits were limited in older adolescents. Several studies have observed that the time elapsed since treatment acts as a moderating factor: the sooner the interventions are implemented after the end of treatment, the stronger the results. Similarly, psychosocial interventions were more effective when there was consistent parental involvement and when the interventions took place in environments that supported skill maintenance. It should also be noted that interventions that incorporate elements of empowerment and family cohesion have more sustainable benefits.

### 3.6. Age-Specific Response Patterns

The effect of interventions varied systematically depending on parameters such as age at the time of intervention, time since completion of treatment, and degree of parental involvement. Interventions implemented within 12–24 months of the end of treatment had a stronger effect, while active family involvement was associated with longer-lasting gains. The analysis of the studies reveals clear developmental differences. Preschool children respond better to emotional regulation and play interventions, while school-age children show greater improvement in social skills and self-regulation interventions. Adolescents, on the other hand, benefit most from interventions that focus on autonomy, identity, and stress management. This differentiation confirms the importance of developing interventions that respond to the characteristics of each age group.

## 4. Discussion

The findings of this review indicate that childhood cancer survivors exhibit a dual vulnerability profile: on the one hand, cognitive difficulties in executive functions and, on the other hand, an increased psychosocial burden that affects integration and daily functioning [78]. In this review, risk factors are conceptualized as characteristics or conditions associated with increased vulnerability to adverse cognitive or psychosocial outcomes, whereas protective factors refer to processes or resources associated with improved adjustment or buffering of cancer-related stress. In the context of the present review, risk factors are defined as individual, relational, or contextual characteristics associated with increased vulnerability to adverse cognitive, emotional, or social outcomes following pediatric cancer. Protective factors, in contrast, refer to processes or emotional resources that buffer stress, support adaptive functioning, and promote resilience across development. In several instances, variables are discussed as protective based on the theoretical targets of interventions rather than on demonstrated longitudinal mediation effects. In most cases, developmentally tailored interventions were compared to standard care, waitlist conditions, or minimal-support controls, rather than to alternative developmentally mismatched interventions. This dual pattern was consistently observed across studies, regardless of intervention type or sample characteristics, suggesting a structural rather than symptom-based vulnerability. The coincidence of these two axes makes one-dimensional interventions less effective and highlights the need for multi-level support [79]. It is important to note that the majority of studies did not include direct comparisons between developmentally distinct intervention models. Rather, interventions tailored to specific developmental stages were most often compared to standard care, waitlist controls, or minimal psychosocial support. As such, conclusions regarding the greater effectiveness of developmentally informed interventions reflect their consistent benefits within age-appropriate contexts, rather than head-to-head superiority over alternative intervention approaches.

Work showing that parental sensory sensitivity predicts children’s visual scanning of emotional stimuli strengthens the argument for including parental characteristics in psychosocial risk assessments in line with PPPHM recommendations [80]. Combined cognitive–psychosocial interventions appear to have a stronger and more stable overall effect on the daily functioning of children and adolescents who are cancer survivors, particularly when implemented over multiple sessions and accompanied by structured follow-up [81]. Strengthening executive functions such as attention, working memory, and cognitive control allows children to apply stress coping strategies more effectively, organize their school and social activities, and adapt to the demands of everyday life [82].

At the same time, psychosocial interventions improve self-regulation, enhance social integration, and promote self-esteem, acting as protective factors against stress, isolation, and emotional difficulties [83]. This two-way relationship between cognitive and emotional development creates a unified framework for strengthening resilience: cognitive skills act as an “infrastructure” that facilitates the implementation of psychosocial strategies, while improved emotional well-being is associated with more consistent cognitive engagement and better social adjustment [84]. Difficulties in hot executive functioning, as reviewed by Kouklari, Christou & Tsermentseli underscore how emotional decision-making capacities can shape children’s adaptation to challenging medical contexts [85,86]. As a result, integrated interventions are not limited to improving individual skills but promote holistic development that enhances the autonomy, functionality, and long-term mental resilience of children and adolescents after cancer [87,88]. Developmental differentiation, therefore, suggests that providing uniform or uniform interventions is ineffective: only interventions that take into account the neurocognitive, emotional, and social capacities of each age group can effectively strengthen resilience and promote long-term adjustment in children and adolescents after cancer [89,90].

The practical application of the findings highlights that interventions for childhood and adolescent cancer survivors must be individually tailored and aligned with their actual developmental needs and the environment in which they live [91,92]. At the clinical level, this means that health professionals must take into account not only the child’s cognitive profile but also factors such as their mental well-being, treatment history, family dynamics, and social relationships [93]. Involving parents in the intervention process is a key component, as they provide emotional security, reinforce coping strategies, and help generalize therapeutic outcomes to everyday life. In an educational setting, collaboration with schools is equally critical: providing educators with structured information on the cognitive and psychosocial challenges of survivors enables more realistic academic expectations and inclusive classroom practices [94]. In addition, the creation of multidisciplinary teams ensures that interventions are not fragmented but coordinated and long-term. Finally, ongoing monitoring after treatment completion is crucial, as cognitive and psychosocial difficulties may emerge or intensify over time; therefore, current evidence supports the conceptualization of support as a longitudinal process, extending beyond treatment completion to accommodate evolving cognitive and psychosocial needs [95].

The existing literature on cognitive and psychosocial interventions in pediatric cancer survivors has significant limitations that affect the reliability and generalizability of its conclusions [96]. First, many studies are based on small samples of participants, which reduces statistical power and makes it difficult to draw clear conclusions for larger populations. In addition, there is considerable heterogeneity in terms of cancer type, intensity, and duration of treatments, age at diagnosis, and time since completion of treatment; these factors significantly influence both cognitive and psychosocial outcomes, making it difficult to compare studies [97]. Another limitation concerns the time horizon of research evaluations: most interventions are measured immediately after completion, without long-term follow-up that could reveal the duration of the effects or the emergence of new difficulties in later developmental stages. At the same time, effectiveness assessments are often based on laboratory tests or questionnaires rather than indicators of actual daily functioning, limiting understanding of the impact of interventions on the qualitative improvement of children’s lives [98]. Finally, in many designs, factors related to the family and socioeconomic environment are not fully taken into account, even though they are decisive for resilience and access to support services. Moreover, substantial methodological variation in assessment tools complicates cross-study synthesis, as different studies operationalize resilience, cognitive performance, or psychosocial adaptation in non-equivalent ways. The coexistence of the above limitations suggests that the current findings, although encouraging, should be interpreted with caution and reinforced by more rigorous, long-term, and multifactorial research protocols [99]. Although the majority of studies lacked long-term follow-up assessments, a small number of interventions that included follow-up periods extending beyond six months suggested that gains in emotional regulation, coping skills, and social adjustment may be partially maintained over time. These findings, although limited, underscore the potential long-term value of early psychosocial intervention while also highlighting the need for more longitudinal research to better understand the durability of intervention effects.

From a clinical perspective, the findings of this review highlight the importance of aligning psychosocial interventions with the developmental stage of the child or adolescent. Clinicians are encouraged to select age-appropriate interventions that integrate cognitive, emotional, and social targets, actively involve families when feasible, and extend support beyond the acute treatment phase. Additionally, ongoing monitoring of psychosocial functioning across survivorship is recommended, as difficulties may emerge or intensify over time. Implementing developmentally sensitive, flexible intervention models may enhance resilience and long-term adjustment in pediatric cancer survivors.

## 5. Conclusions

This review underscores that the development and implementation of psychosocial interventions in pediatric oncology must be grounded in a clear developmental rationale. Children and adolescents affected by cancer experience complex and evolving cognitive, emotional, and social challenges that differ substantially across developmental stages [100]. Accordingly, interventions that are developmentally calibrated appear more effective in promoting adaptive functioning and long-term resilience. In particular, models integrating cognitive and psychosocial components address both neurocognitive vulnerabilities associated with cancer treatment and the emotional and social demands of survivorship. Active family involvement and collaboration with educational and clinical systems further enhance intervention effectiveness and facilitate the transfer of acquired skills to everyday contexts, consistent with evidence from pediatric chronic illness and oncology research emphasizing the central role of family and coping processes in psychosocial adaptation [101,102].

Despite these encouraging findings, the existing literature remains constrained by methodological limitations, including small sample sizes, heterogeneous populations, and limited longitudinal follow-up [103,104]. Consequently, future research should adopt more rigorous designs, incorporate developmental trajectories over time, and systematically examine cultural and contextual moderators of intervention outcomes. Overall, the present review highlights that integrating a developmental perspective into psychosocial care is not merely complementary but fundamental to supporting resilience, functional adaptation, and long-term well-being among pediatric cancer survivors.

Emerging evidence from developmental neuroscience offers additional, albeit still preliminary, insights into the mechanisms that may shape individual differences in intervention responsiveness. Studies demonstrating parent–child covariation in attentional and emotional processing suggest that dyadic regulatory processes may influence children’s emotional and behavioral outcomes, thereby informing the design of caregiver-inclusive intervention models [105]. Similarly, findings indicating that parental sensory processing sensitivity is associated with children’s emotion-processing patterns highlight the potential relevance of caregiver neurocognitive characteristics in psychosocial risk assessment and intervention tailoring [106,107]. However, the current evidence base is still emerging, with many studies relying on correlational designs or non-oncological samples, suggesting that the role of parental neurocognitive processes in shaping intervention outcomes in pediatric oncology warrants further investigation.

Within pediatric oncology, specifically, the present review demonstrates that intervention effectiveness is most consistently associated with developmental timing, clinical context, and psychosocial targets rather than with specific neurocognitive mechanisms alone. Age-appropriate interventions—such as play-based approaches in early childhood, cognitive–behavioral and school-focused strategies in middle childhood, and autonomy- and identity-oriented interventions in adolescence—are more reliably linked to improvements in emotional regulation, social adjustment, and functional outcomes. These findings align with broader evidence showing that psychological interventions in pediatric populations yield meaningful improvements when tailored to developmental and clinical characteristics [108]. From a clinical perspective, the results emphasize the need to embed psychosocial interventions within developmentally sensitive care pathways that address the unique cognitive and emotional sequelae of cancer and its treatment, including the risk of persistent stress-related symptoms in survivors and their families [109].

Neurocognitive and dyadic processes may therefore be conceptualized as potential moderators or explanatory frameworks that complement, rather than replace, developmental and psychosocial models of intervention effectiveness. For example, evidence on emotion–feeling differentiation in parent–child dyads and individual differences in environmental sensitivity suggests that neurocognitive traits may influence children’s responsiveness to psychosocial interventions [110,111]. Similarly, advances in assistive and digital technologies aimed at reducing neurophysiological stress and enhancing cognitive engagement point toward innovative avenues for personalized intervention delivery in pediatric oncology settings [112,113]. These emerging approaches hold promise for refining intervention models but require further empirical validation within oncology-specific contexts.

Taken together, the findings of this review support the conclusion that psychosocial interventions in pediatric oncology are most effective when they are developmentally tailored, clinically integrated, and contextually grounded. Developmental timing and psychosocial targeting remain the most robust and clinically actionable determinants of intervention success, while neurocognitive and dyadic mechanisms represent promising directions for future research rather than established causal pathways. Future studies should therefore prioritize longitudinal, oncology-focused investigations that integrate developmental, familial, and neurocognitive perspectives to advance more precise, developmentally informed, and evidence-based psychosocial care for children and adolescents with cancer.

## Figures and Tables

**Table 1:** Types of interventions and developmental goals for children and adolescents after cancer.

Intervention Type	Developmental Focus	Core Strategies	Main Outcomes
**Cognitive Interventions**	School-age children & adolescents	Computerized training (Cogmed), Working memory enhancement programs [57,58]	Improvement in executive functions, attention, and working memory, Limited generalization to everyday functioning, Greater effectiveness in long-term programs and at younger ages [59,60]
**Psychosocial Interventions—Infancy & Toddlerhood (0–2)**	Need for secure relationships and emotional stability [37]	Parent guidance, Emotional regulation support, Strengthening secure attachment [61,62]	Stable emotional foundation, strengthening early resilience, reducing maladaptive reactions [63]
**Psychosocial Interventions—Early Childhood (3–5)**	Development of emotional regulation & social skills	Play therapy, Social skills activities, Individual counseling, Active parental involvement [64]	Strengthening coping mechanisms, emotional self-regulation, positive social interaction [65,66]
**Psychosocial Interventions—Middle Childhood (6–11)**	Self-regulation, social participation, problem solving	Social skills groups, Counseling, Educational activities for autonomy [67]	Improving self-management, adapting to school, developing resilience through social empowerment [68,69]
**Psychosocial Interventions—Adolescence (12–18)**	Autonomy, social inclusion, stress management	Mindfulness, Individual counseling, Support groups, Stress management techniques [70,71]	Reduction in emotional difficulties, enhancement of independence, social adjustment, and resilience [72,73]
**Combined Cognitive–Psychosocial Interventions**	All ages, with development-compatible design	Combination of executive functions (memory, attention) and psychosocial skills (self-regulation, participation), Intervention according to the developmental stage [74,75]	Systematically better daily functioning, adaptation to the school/social context, increased resilience, enhanced autonomy and social integration [76,77]

## Data Availability

No new data were created or analyzed in this study. Data sharing is not applicable to this article.
